# Design and construction of an optimized transmit/receive hybrid birdcage resonator to improve full body images of medium-sized animals in 7T scanner

**DOI:** 10.1371/journal.pone.0192035

**Published:** 2018-02-01

**Authors:** Nibardo Lopez Rios, Philippe Pouliot, Konstantinos Papoutsis, Alexandru Foias, Nikola Stikov, Frédéric Lesage, Mathieu Dehaes, Julien Cohen-Adad

**Affiliations:** 1 Department of Electrical Engineering, Ecole Polytechnique Montreal, Montreal, QC, Canada; 2 Sainte-Justine Hospital University Center, Montreal, Canada; 3 Medical Biophysics Center, University of Oriente, Santiago de Cuba, Cuba; 4 Research Center, Montreal Heart Institute, Montreal, QC, Canada; 5 FMRIB Centre, Nuffield Department of Clinical Neurosciences, University of Oxford, Oxford, United Kingdom; 6 Department of Radiology, Radio-oncology and Nuclear Medicine, University of Montreal, Montreal, Canada; 7 Institute of Biomedical Engineering, University of Montreal, Montreal, Canada; 8 Functional Neuroimaging Unit, CRIUGM, University of Montreal, Montreal, QC, Canada; University of Pennsylvania Perelman School of Medicine, UNITED STATES

## Abstract

The purpose of this work was to develop an optimized transmit/receive birdcage coil to extend the possibilities of a 7T preclinical MRI system to conduct improved full body imaging in medium-sized animals, such as large New Zealand rabbits. The coil was designed by combining calculation and electromagnetic simulation tools. The construction was based on precise mechanical design and careful building practice. A 16-leg, 20 cm long, 16 cm inner diameter, shielded quadrature hybrid structure was selected. Coil parameters were assessed on the bench and images were acquired on phantoms and rabbits. The results were compared to simulations and data obtained with an available commercial coil. An inexpensive assembly with an increase of 2 cm in useful inner diameter and 50 Ω matching with larger animals was achieved. A reduction in radiofrequency (RF) power demand of 31.8%, an improvement in image uniformity of 18.5 percentage points and an increase in signal-to-noise ratio of up to 42.2% were revealed by phantom image acquisitions, which was confirmed by in vivo studies. In conclusion, the proposed coil extended the possibilities of a preclinical 7T system as it improved image studies in relatively large animals by reducing the RF power demand, and increasing image uniformity and signal-to-noise ratio. Shorter scans and time under anesthesia or reduced RF exposure, resulting in better images and lower animal health risk during in vivo experiments, were achieved.

## Introduction

Rabbits are medium-sized animals of importance for cardiac research due to some similarity of their hearts to those of humans and their lower cost compared to larger animals [1]. However, they hardly fit in most preclinical scanners and nearly all in vivo MRI is currently performed in clinical scanners, leading to higher study costs and sub-optimal imaging protocols [2–5].

For example, the locally installed Agilent (Varian) 7T scanner (Santa Clara, California, US) has a 21 cm bore, which is reduced to 14 cm by the largest available radiofrequency (RF) coil: a quadrature birdcage made by the same manufacturer for a loading range that includes rabbits. This type of commercial volume coil is usually optimized to fit the average sample of a selected loading range, but they can be impractical when more space is required to accommodate larger animals, such as obese rabbits of more than 4 kg used for cardiac research. For these particular studies, the available commercial coil shows reduced sensitivity and increased RF power demand due to its limited tuning/matching range.

This work presents the development of an optimized transmit/receive birdcage with extended space, increased image uniformity and higher signal-to-noise ratio (SNR). These improvements expanded the application range of the aforementioned scanner to medium-sized animals, such as large New Zealand rabbits, which cannot be optimally studied with the commercial coil.

## Materials and methods

### Design and construction

A quadrature transmit/receive birdcage was designed for an Agilent (Varian) 7T scanner with a magnet bore being 170 cm long and 21 cm in diameter. A hybrid structure was selected as it is deemed the most suitable for the specified Larmor frequency (*f*_*0*_ = 299.5 MHz), dimensions and intended subjects [6].

Preliminary design and component values were obtained from the on-line Java Circular Birdcage Builder (Penn State Hershey College of Medicine, Pennsylvania, US) [7]. The results were transferred to FEKO (Altair Development S.A. (Pty) Ltd, South Africa), where a realistic and completely reconfigurable 3D model of the coil was created. The shield, the interfacing circuits, and the loading were included. Cylindrical structures made of biological tissues, liquids and solids from the FEKO media library were used to load the coil during the simulations. A combination of the method of moments and the finite element method (MoM/FEM) was selected, given its proven accuracy to simulate MRI coils [8, 9].

RO4350B laminate (Rogers, Connecticut, US) was selected as the construction material for the resonator and the shield [10, 11]. This material is a two-layer printed circuit board (PCB) with copper cladding of 35 μm (1oz). The substrate thickness was 0.25 mm (10 mils) for the resonator and 0.1 mm (0.4 mils) for the shield. Parallel plate capacitors were created along the legs of the resonator by overlapping the edges of seven PCB sections [12, 13].

To reduce computation time in FEKO during initial simulation steps, the thin PCB substrate of the resonator was replaced by air and the overlapped area of the parallel plate capacitor was increased to compensate the change of capacitance. With the same purpose, a custom mesh was selected. 1 to 2 cm was the length of the triangle/tetrahedral edges and 0.1 to 0.2 cm was the wire segment length. In the final design stages the mesh dimensions were also reduced and local mesh refinement was applied, which considerably increased simulation time. In some simulations, such as those run to optimize the shield, the parallel plate capacitors were replaced by loads, containing capacitance and equivalent series resistance, to reduce simulation time.

Resonators having 16 and 24 legs, and lengths from 15 cm to 23 cm were evaluated. A 16-leg, 18.7 cm long structure (between the centers of the end rings) was selected after an iterative design and simulation process. These parameters fulfilled the requirements of achieving a suitable magnetic field distribution over a region of interest that covers the abdominal-thoracic region of rabbits used for obesity studies related to cardiac diseases. The legs width was set to 1.27 cm resulting in a conductors-to-spaces ratio of approximately 2/3 on the resonator structure. The same width was selected for the end rings. The total leg capacitance was split into six capacitors to reduce the electric length of the legs sections to 2.67 cm. This is less than lambda/20 (at *f*_*0*_) and smaller than the average distance between the resonator surface and the animal body [12, 14]. Each capacitor has 6 pF resulting from an overlapped area of 1.277 x 0.325 cm. End ring capacitances are made of two surface mount capacitors connected in parallel (20 + 15 pF) to reduce the total equivalent series resistance (ESR) and distribute the current flow, which reduces the temperature rise and capacitance change. The resonator diameter is 16.6 cm, which is reduced by the supporting structure to a useful diameter of 16 cm. This is 2 cm larger than the commercial coil whose inner diameter (externally measured) is 14 cm. The length of the commercial coil is approximately 18 cm and the number of legs is 16, as estimated from acquired images.

**Fig 1:**
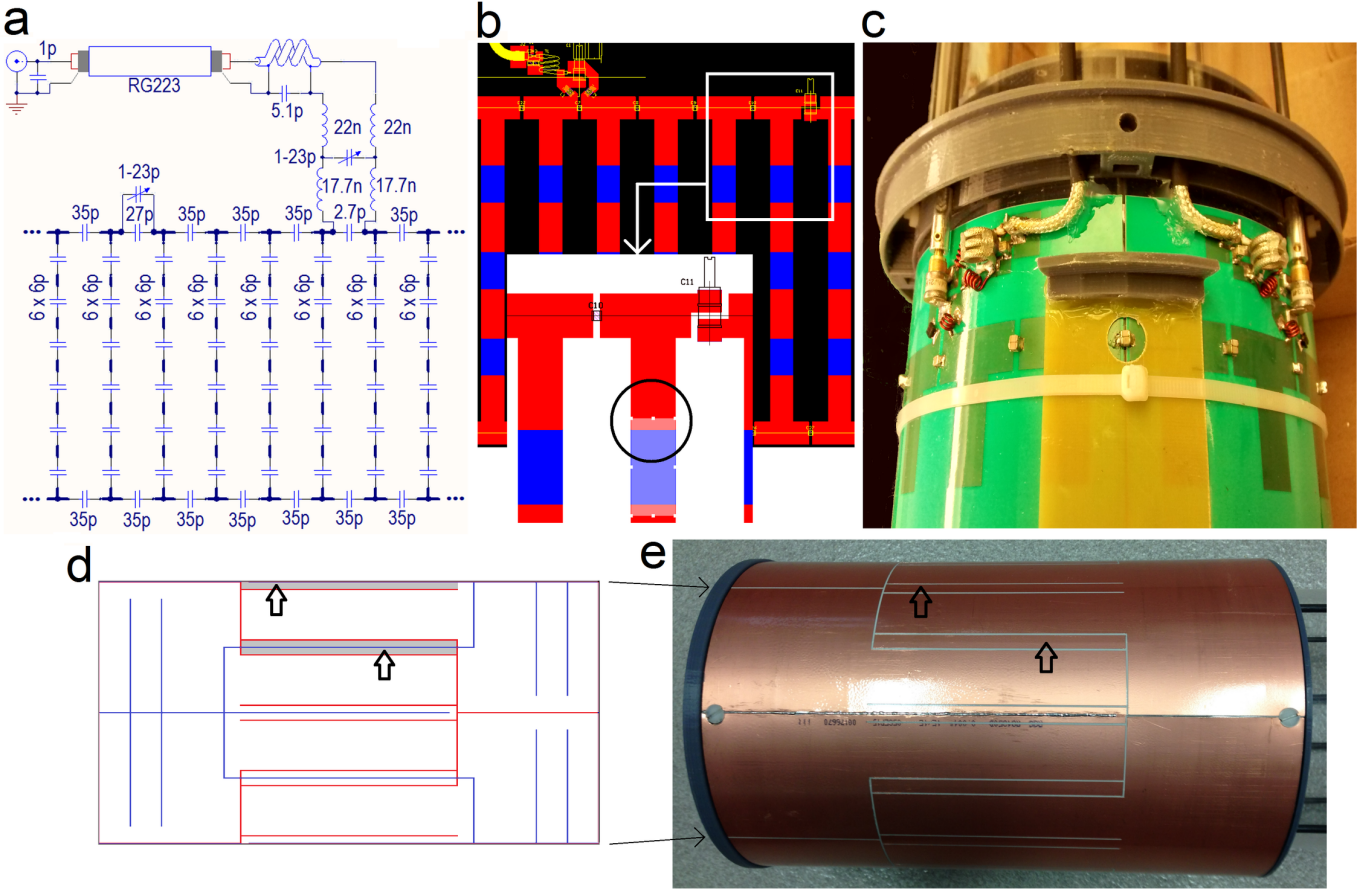
Electrical design of the coil. **(a)** Schematic design with actual component values. The interfacing circuit of channel ***Q*** and the tuning capacitor of channel ***I*** (1-23p) are shown. The bold lines represent copper traces conforming the resonator. **(b)** PCB design with zoomed panel showing one parallel plate capacitor in transparency (black circle). The blue and red regions represent the copper sections on the inner and outer surfaces, respectively. **(c)** Picture of the resonator showing interfacing circuits (***Q*** and ***I***) and cable traps. **(d)** PCB design of the shield (one of four sections). The blue and red traces represent the gaps on the inner and outer copper surfaces, respectively. **(e)** Picture showing outer surface of the shield. The arrows show copper regions created to improve RF isolation.

Fig 1A shows a section of the electrical design. The matching transformer is a balanced *T* network made of series inductors and one multi-turn variable capacitor (NMAJ25HVE, Voltronics, Salisbury, MD, US) used as a matching control element. A cable trap was selected as the balanced-unbalanced transformer for each channel and their positions and orientations were chosen to minimize mutual coupling. A section of the layout is presented in Fig 1B and the exposed resonator in Fig 1C.

The shield is based on two concentric cylindrical arrays made of PCB copper sections, separated by 1 mm gaps, that create a capacitive network, as presented before [10, 11]. Structural details were modified to improve shielding and avoid self-resonances near *f*_*0*_. Other modifications, such as some conveniently placed copper regions (8 x 130 mm), were introduced to increase the shielding effect in the RF range without affecting the response to field gradients (see arrows in Fig 1D and 1E). The diameter of the shield was maximized according to the magnet bore.

The initial simulations of the shield were made in a flat version, which reduced computing time as the multilayer substrate feature included in FEKO was used. Two magnetic field loops were modeled and placed on each side of the flat shield version to assess the isolation over the whole area by measuring S_21_ between them. To confirm the results, a prototype section was subsequently built and assessed on the bench by the same method. The same process was performed for the cylindrical final version.

**Fig 2:**
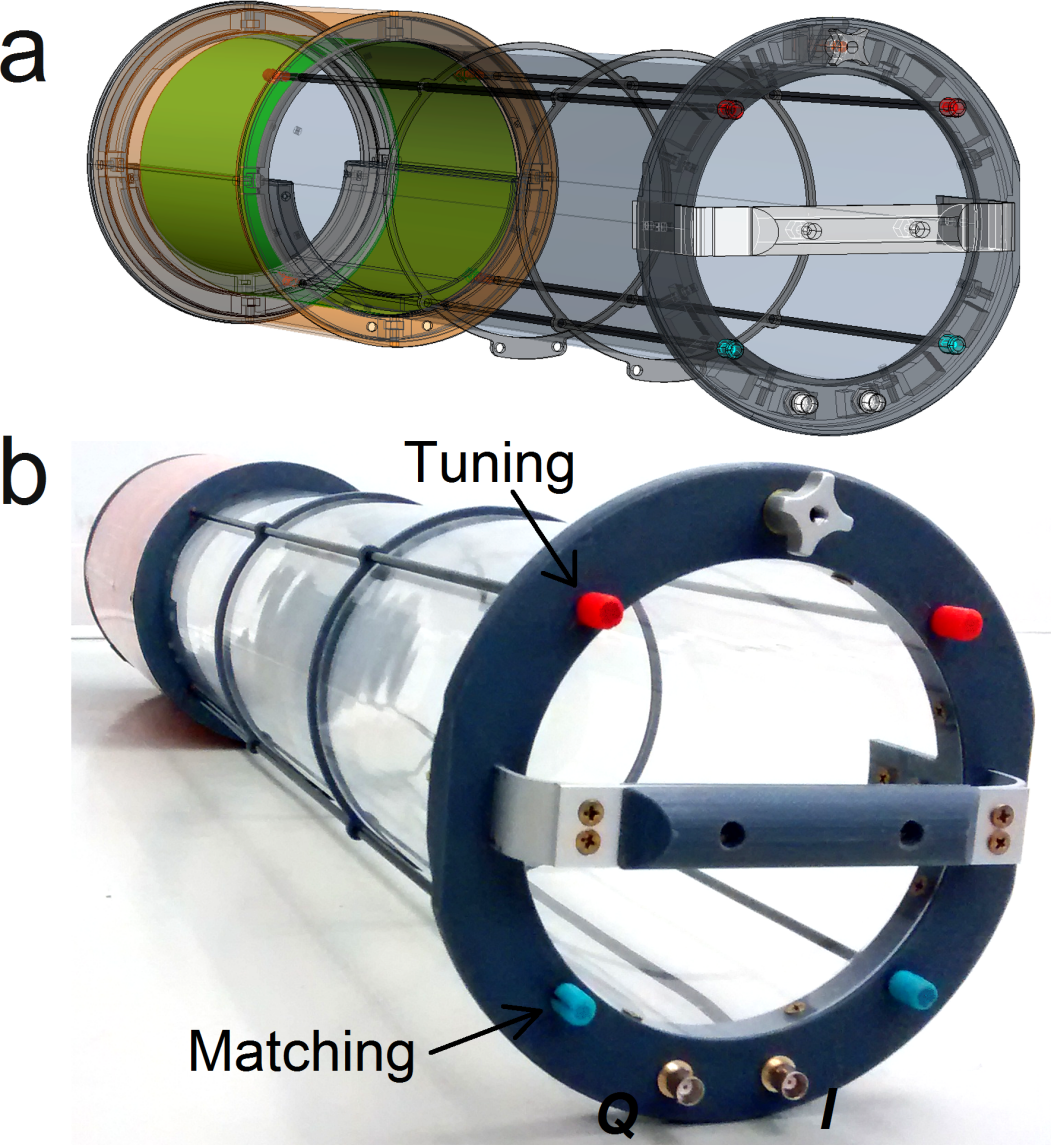
Mechanical design of the coil. **(a)** CAD design and **(b)** picture of the coil assembly. ***Q*** and ***I*** are BNC terminals connected to the cable traps by RG-223 coaxial cables. Tuning and matching can be adjusted for each channel by means of the red and blue knobs attached to fiberglass rods extensions of the corresponding capacitors. A handle and a simple holding mechanism facilitate the installation and fastening of the coil into the magnet bore.

The resonator was built by wrapping the PCB around an acrylic glass tube to guarantee the cylindrical symmetry [10, 11]. The shield was made by soldering four identical PCB sections and wrapping it around a 30 cm long Plexiglas tube with an external diameter of 20.3 cm as shown in Fig 1D and 1E [10]. All mechanical parts are supported on the Plexiglas tube (length = 101.8 cm, outer diameter = 16.6 cm, thickness = 3 mm) used as a holder for the resonator. AutoCAD 2011 (Autodesk, Inc., San Rafael, CA, US) was used to design the coil (see Fig 2A) and most of the parts were printed in polylactic acid (PLA) material with a Big Builder 3D printer (Code-p West B. V., The Netherlands). The constructed coil is completely shown in Fig 2B.

Two different phantoms were created. First, a 2-liter cylindrical bottle (diameter = 11 cm, length = 20 cm), filled with a solution simulating an average biological tissue [15]. An on-line tool (https://amri.ninds.nih.gov/cgi-bin/phantomrecipe) was used to obtain an electrical conductivity of 0.7 S/m and a relative permittivity of 50 by mixing the following ingredients: NaCl (74.3g), sugar (1494g) and water (1062g) [16]. The final weight of the phantom was 2.8 kg and its loading effect was evaluated on the bench and the scanner to ensure the equivalence with the body parts of a large rabbit that fit in the coil during the studies. The coil was adjusted and tested on the bench with this load. The other phantom was a mineral oil cylindrical container with a diameter of 12 cm and a length of 20 cm.

### Bench tests

The parameters of the parallel plate capacitors, the efficiency of the shield, the S-parameters and the quality factors of the coil were measured with a network analyzer (Agilent E5061B). Tuning, matching and isolation between channels were evaluated with the water/NaCl/sugar phantoms. The unloaded and loaded quality factors (Q) were measured to evaluate the noise dominance in the coil. The frequency shift between both conditions was noted as well. The loaded Q was tested with only two thirds of the water/NaCl/sugar phantom inside the coil to allow the placement of the measuring probes [17].

RF magnetic field (*B*_*1*_) homogeneity was assessed by exciting the coil in quadrature with a 90-degree hybrid coupler (R432.171.000, RADIALL S.A., Paris, France) and measuring S_21_ with a magnetic field probe which was positioned inside the coil in discrete sites of a coaxial cylindrical region (diameter = 13 cm, length = 14 cm) [12]. The balance between individual linearly polarized magnetic fields was previously verified in a similar way. The effectivity of the shield was also tested by reading S_11_ and S_22_ while its external surface was explored with a 6-cm wire loop tuned at the Larmor frequency with a variable capacitor. The isolation values were measured with two parallel-placed magnetic field probes, as explained above.

### MRI tests

Images acquired with the proposed coil and the available commercial coil were compared. Both coils were tuned and matched as well as possible while in the scanner using a portable network analyzer (Agilent N9923A FieldFox) before image acquisitions.

#### Phantoms

Both phantoms were scanned. First, the mineral oil cylindrical phantom was scanned with the main purpose of assessing *B*_*1*_ homogeneity. The selected sequence was a Gradient Echo Multi Slice (GEMS) with the following parameters: Pulse Repetition Time (TR) = 50 ms, Echo Time (TE) = 5 ms, Acquisition Time (AT) = 12 s, Field of View (FOV) = 13 cm, matrix = 256 x 256, slice thickness = 2 mm, RF pulse shape = gauss and RF pulse length (180^o^) = 2 ms. The phantom was directly placed in the cradle and not moved between acquisitions.

Afterward, Fast Spin Echo Multiple Slice (FSEMS) axial images were acquired with the water/NaCl/sugar phantom to evaluate the image SNR. The parameters of this sequence were: TR = 1 s, TE = 13 ms, AT = 64 s, FOV = 13 cm, matrix = 512 x 512, slice thickness = 2 mm, RF pulse shape = gauss, RF pulse length (180°) = 3.2 ms. The phantom was coaxially placed in the coil by means of foam supports.

SNR maps were used to compare the coils. They were calculated by dividing each pixel by the standard deviation of the noise within a selected region in the background corresponding to a corner of one 10^th^ by one 10^th^ of the image dimensions [18]. Notice that the two quadrature channels of the coil are connected to a single receiver channel in the scanner through a 90-degree hybrid power combiner. The image uniformity was calculated by following the method ACR MR Accreditation Program (ACR-MRAP) Uniformity, described in [19]. This method reduces the influence of image SNR on the analysis. No pre-processing of the image was performed. The averages of the intensities inside subregions of interest of 10x10 pixels, placed in areas of the image with the highest and lowest intensity levels, were computed to subsequently determine the image non-uniformity.

#### Rabbits

Established animal care recommendations were followed during all in vivo studies performed with both coils and the procedures were approved by the Animal Research Ethics Committee of the institution (Montreal Heart Institute Animal Ethical Committee). A group of over 120 studies on rabbits performed with both coils, both healthy and with diseases, was used as a database to compare the coils. Sixteen were selected at random to collect the RF power required to generate a 90^o^ pulse during the initial calibration. This power can be used to assess how well the coils can be matched to the RF power amplifier while loaded with a large sample. Eight studies were chosen for each coil according to the following criteria: the animals were all New Zealand healthy white rabbits in a narrow weight range (weights = 2.9–3.5 kg, average = 3.2 kg for each group). Abdomen imaging of these rabbits was performed with True Fast Imaging with Steady State Precession (TrueFISP) [20]. The parameters of the sequence were: FOV = 19.2 x 12.8 x 12.8 cm, matrix size 144 x 96 x 96, with TR in the range of 4.8 to 6.0 ms, and imaging time 19–26 minutes, depending in part on the rabbit weight. Some images from these sixteen studies were used to verify the performance of the coils with non-homogeneous loads.

In addition, one healthy rabbit (weight = 3.2 kg) was scanned with both coils to compare image quality. This rabbit was positioned in the cradle and not moved between experiments, and the time lapse between both acquisitions was 16 minutes. For this rabbit, a spoiled-gradient recalled echo multi-slice was used (SPGR GEMS, TR = 50 ms, TE = 5 ms, FOV = 13 cm, matrix = 256 x 256, slice thickness = 2 mm, RF pulse shape = gauss and RF pulse length (180^o^) = 2 ms). Rabbits were tranquilized with 100uL/kg Atravet and put in an induction cage. After 10–15 minutes, anesthesia induction was started at 5% isoflurane. Once the rabbits were unresponsive, they were moved to the imaging bed and maintained under 2.5–3.0% isoflurane as required to maintain a respiratory rate of 25–35 BPM. Temperature was maintained at 39.0 C with air flow and heart rate was monitored with a pulse oximeter attached to a front paw (Small Animal Instruments, New York).

## Results

### Simulations

**Fig 3:**
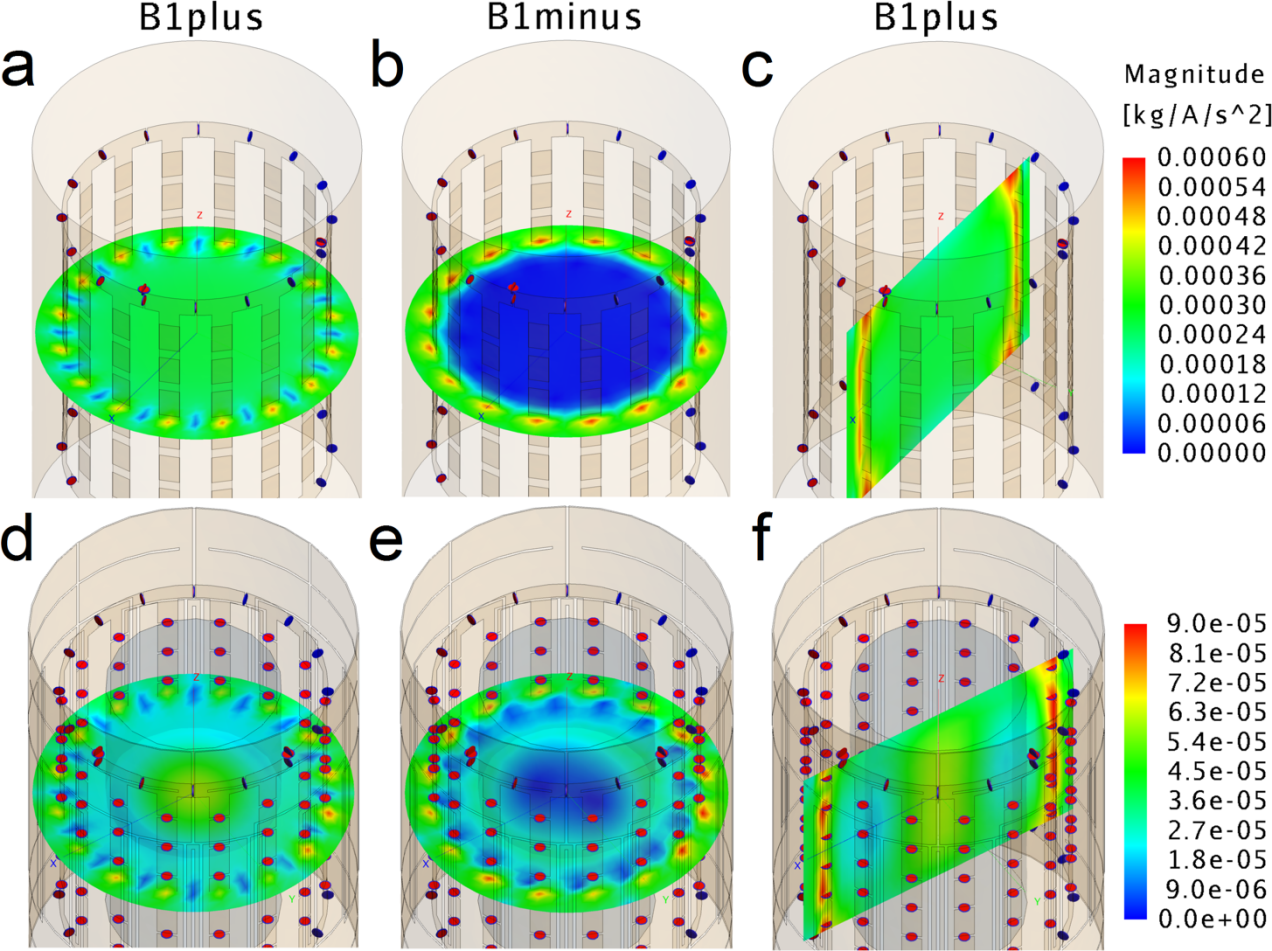
Magnetic field magnitude maps generated at the center of the birdcage. **(a)** *B*_*1*_^+^ axial, **(b)** *B*_*1*_^-^ axial and **(c)** *B*_*1*_^+^ sagittal, proving high efficiency and good homogeneity. **(d)** *B*_*1*_^+^ axial, **(e)** *B*_*1*_^-^ axial and **(f)** *B*_*1*_^+^ sagittal obtained from the coil loaded with a cylindrical sample similar to the water/NaCl/sugar phantom (electrical conductivity = 0.7 S/m, relative permittivity = 50, density = 1280 g/dm^3^, diameter = 11 cm and volume = 2000 cm^3^). The input power for each coil channel was 500 W, as 1 kW is the maximum available in the scanner.

The homogeneous *B*_*1*_ magnitude distribution created by the empty coil is shown in Fig 3A, 3B and 3C. Good circular polarization was also obtained since *B*_*1*_^+^ (a) is considerably higher than *B*_*1*_^-^ (b) inside the coil. This result was confirmed by obtaining the expected field distributions in a selected homogeneous cylindrical load at 7 T, as shown in Fig 3D and 3E. The values of S_11_, S_22_ and S_21_ were always below -23 dB reflecting good matching and isolation between quadrature channels.

### Bench tests

The quality factor of the parallel plate capacitors was 454 at 300 MHz, which results in an equivalent series resistance (ESR) of 0.194 Ω. This value produces a total resistance in the coil (18.6 Ω) that is much smaller than the animal loading, which is in the range of 200 to 2000 Ω [17]. Return loss (RL) values below -35 dB were obtained in both terminals by manually tuning and matching the coil with the 2-liter water/NaCl/sugar phantom used as a load. The isolation between channels at *f*_*0*_ was -50.5 dB. Sample noise dominance was obtained with the coil as the unloaded Q was 209.8 and the loaded Q was 25.3, which results in a ratio of 8.3. A frequency shift of 1.8 MHz was observed between both conditions. An S_21_ deviation of less than 1.5 dB for all orientation angles was observed during the *B*_*1*_ homogeneity evaluation. A coil detuning of less than 0.4 MHz was observed when the complete external surface of the shield was explored with the tuned 6cm wire loop. The RF attenuation of the shield, measured at all positions around the end rings with the magnetic field parallel probes, was between -31 dB and -40 dB.

### MRI tests

#### Phantoms

With the water/NaCl/sugar phantom, S_11_ and S_22_ levels of -8.7 and -7.6 dB respectively, were obtained with the commercial coil, whereas -23.6 dB and -21.3 dB were measured in both channels of the proposed coil. A detuning of 1.2 MHz was measured when the proposed coil, loaded with this phantom, was inserted in the gradient tube after being tuned outside the scanner. This result confirmed the good isolation achieved with the proposed shield. A similar value (1.5 MHz) was obtained with the commercial coil.

**Fig 4:**
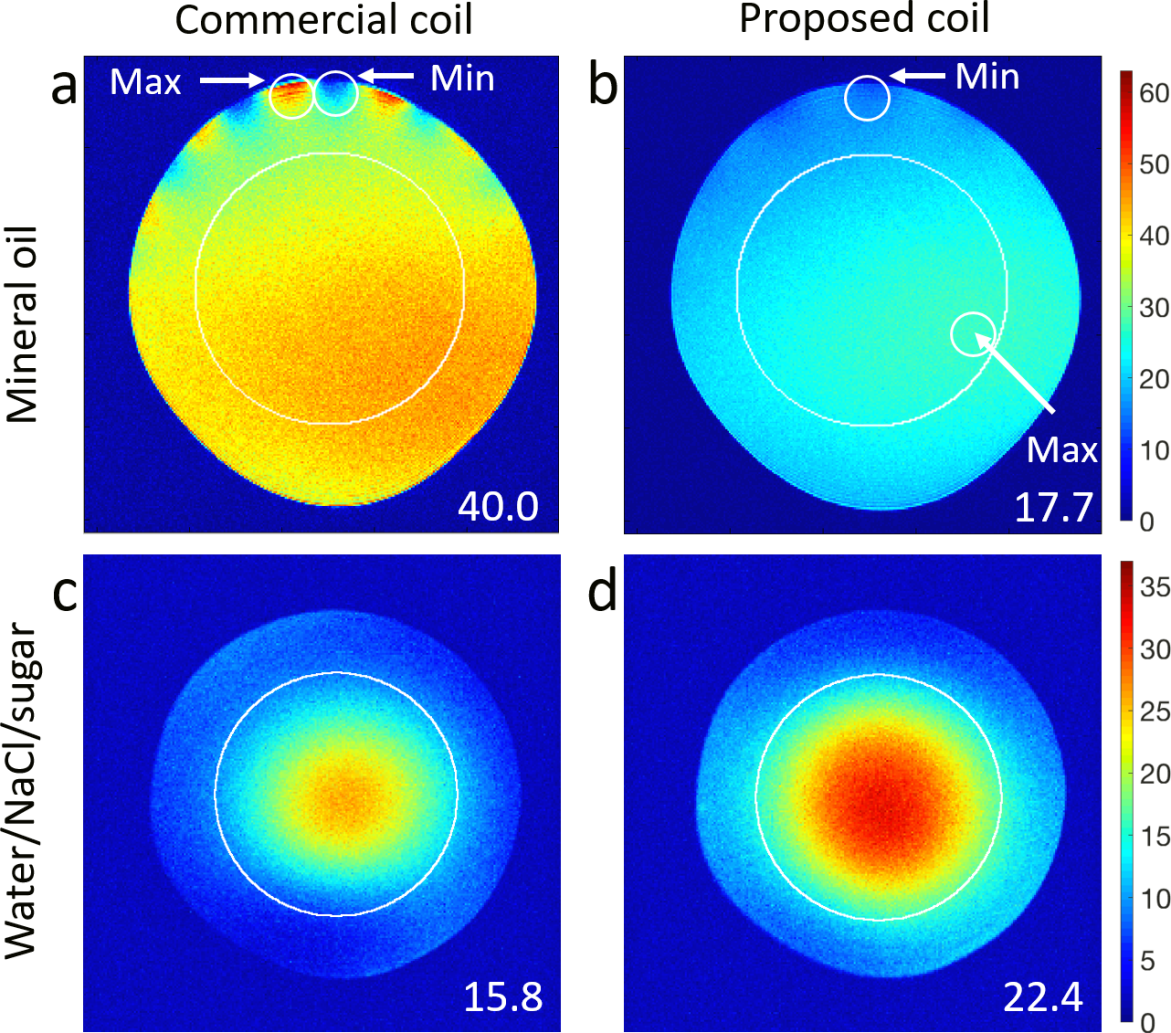
Phantom acquisitions at the isocenter. **(a, b)** SNR maps, computed from the Gradient Echo Multi Slice (GEMS) images of the mineral oil phantom, showing better uniformity and reduced SNR with the proposed coil. The small white circles show the areas with maximum and minimum pixel intensities. The subregions of interest (10x10pixels) used to calculate the uniformity were selected inside these circles. **(c, d)** SNR maps calculated from Fast Spin Echo Multiple Slice images of the water/NaCl/sugar phantom, showing increased SNR (41.8%) with the proposed coil in a selected circular ROI. The numbers in white are the corresponding average SNR computed inside these ROIs.

SNR maps computed from the Gradient Echo images of the mineral oil phantom are presented in Fig 4A and 4B. The percent image uniformity (PIU), calculated from the commercial coil image (a) was 50.1% whereas the proposed coil (b) reported 68.6%, which is an improvement of 18.5 percentage points. However, an average SNR decrease of 44.3% was observed in a circular region with a radius of 2/3 the phantom radius (40.0 for the commercial coil and 17.7 for the proposed coil). The SNR maps calculated from FSEMS axial images of the water/NaCl/sugar phantom are shown in Fig 4C and 4D. The proposed coil exhibited improved field distribution, good agreement with simulations (Fig 3D), and higher SNR. The mean SNR value in the complete phantom image was 42.2% higher (10.9 for the commercial coil and 15.5 for the proposed coil). Inside a circular region of interest having a radius of 2/3 the phantom radius, the mean value of the SNR in was increased by 41.8% (15.8 for the commercial coil and 22.4 for the proposed coil, as shown in the corresponding maps).

### Rabbit abdominal acquisitions

A reduction of 31.8% in average RF power required to generate a 90^o^ pulse was found in the recorded values for the 8+8 rabbits (192.4 W by the commercial coil and 131.2 W by the proposed coil). This power decrease made possible a significant scan time reduction (15–25% for TrueFISP), due to shorter pulse duration allowed, since the coil was typically operated at the maximum power available from the RF power amplifier (1000 W nominally). As a result, the time under anesthesia and the health risks for the rabbits were reduced and the workflow improved.

**Fig 5:**
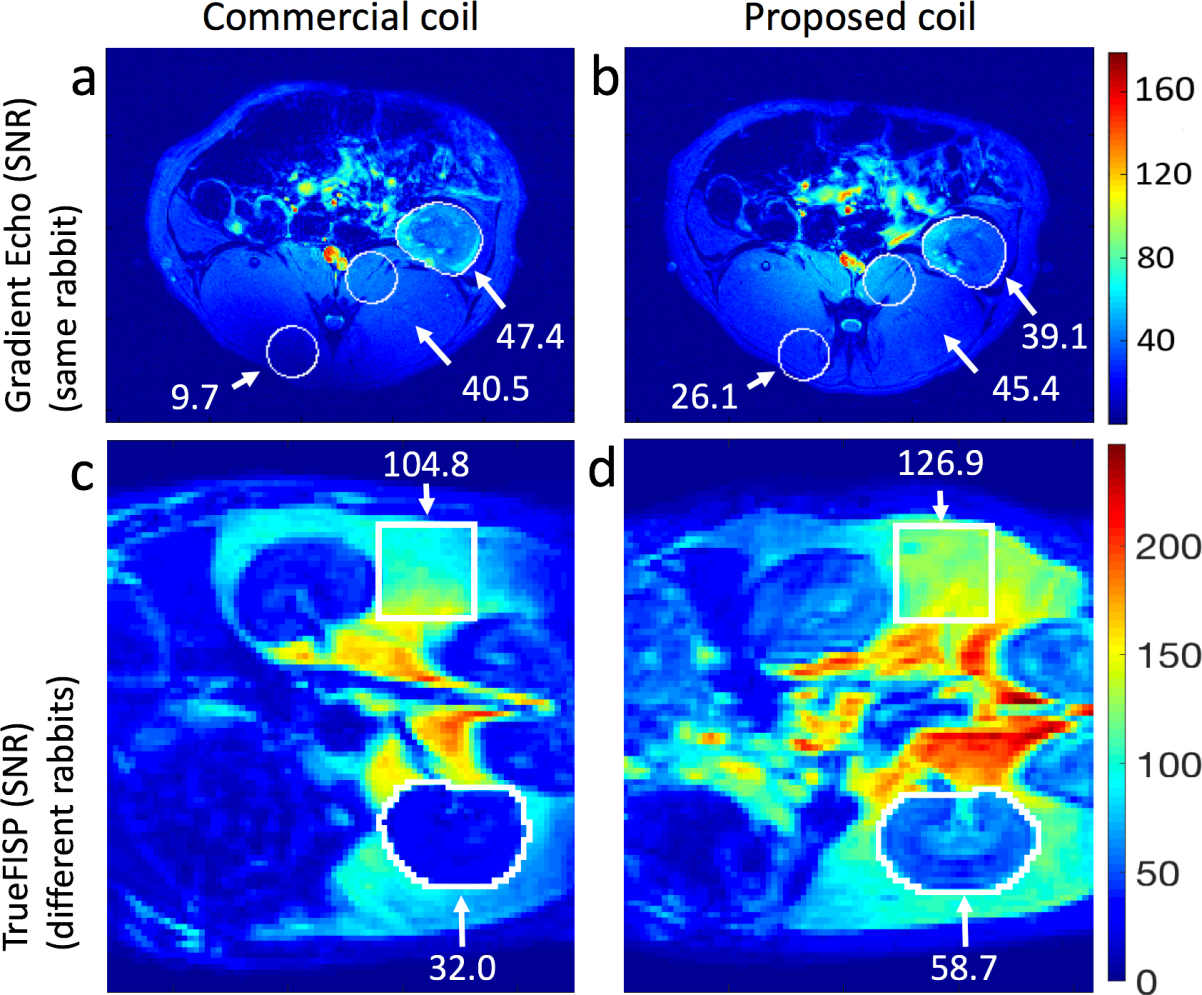
Rabbit acquisitions. **(a, b)** SNR maps computed from Axial Gradient Echo Multi Slice images of the same rabbit acquired with both coils at the isocenter. **(c, d)** Coronal SNR maps calculated from TrueFISP experiments performed on different rabbits. The numbers in white are the mean SNR values calculated in the associated enclosed regions.

SNR maps calculated from Axial Gradient Echo sample images acquired through the abdominal region of the selected healthy rabbit with both coils, are shown in Fig 5A and 5B. They reveal improved image uniformity achieved with the proposed coil. The mean SNR value in the enclosed circular regions, corresponding to muscle, are 12.1% and 169.1% higher in the proposed coil. However, a region of interest selected in one kidney shows a mean SNR reduction of 17.5%.

SNR maps computed from the TrueFISP available images of two different healthy rabbits are displayed in Fig 5C and 5D. The rabbits were selected from both groups considering that they had a similar amount of fat. Local average SNR values are shown in coronal slices acquired at approximately the same position with respect to the kidneys. A higher mean SNR in the fat regions was obtained with the proposed coil. A squared region of interest in that region, where some obesity-related studies are performed, resulted in an increase of 21.0%. The higher SNR in the fat region was also observed in the rabbit scanned with both coils (Fig 5A and 5B). In contrast, an increase of 83.4% was obtained in one kidney with this sequence.

**Fig 6:**
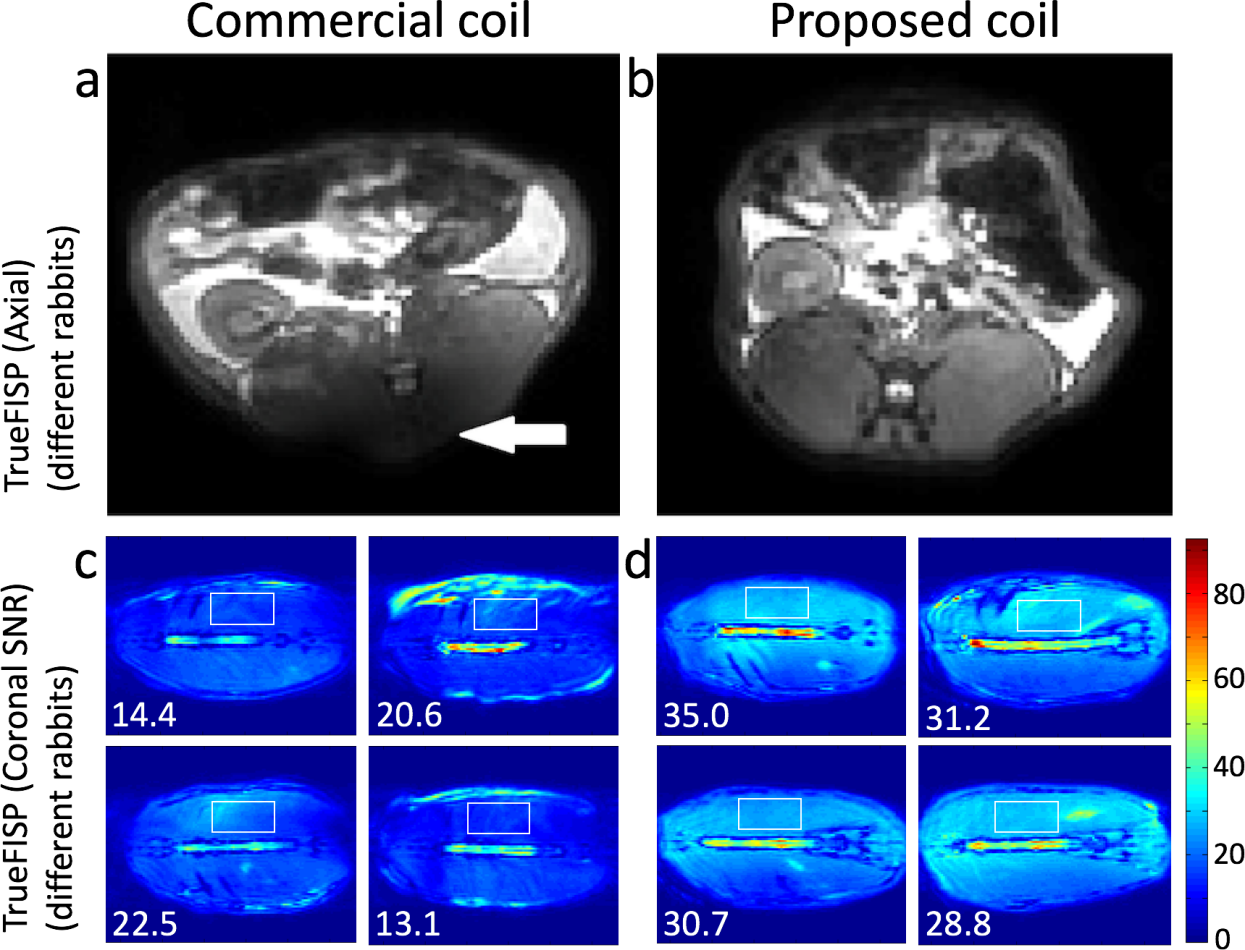
Rabbit acquisitions. **(a, b)** Axial TrueFISP slices of different rabbits showing the improved uniformity achieved with the proposed coil. **(c, d)** Coronal SNR maps with selected rectangular regions of interest and corresponding mean values. The higher increase in SNR is visible at the ends of the coil.

Other images, acquired with the TrueFISP sequence are shown in Fig 6. Better B1 uniformity was also obtained with the proposed coil as the dark region in the lower part of the commercial coil axial images (white arrow in Fig 6A) is not present in the proposed coil image (Fig 6B). In addition, SNR maps computed on coronal slices selected at the level of the spine show an increase in SNR. As can be seen in Fig 6C and 6D, the tissue in this region is more homogeneous (mostly muscle) and more similar among rabbits, which facilitates the comparison. Selected regions in four maps from each group of rabbits resulted in average values of 17.7 and 31.4 for the commercial coil and the proposed coil respectively, which is an increase of 77.4%. A uniform SNR distribution is also observed along the complete coil length.

## Discussion

Some difficulties exist to study medium-sized laboratory animals, such as obese rabbits, in 7T preclinical scanners due to the relatively limited dimensions of the commercial coils and the deterioration of their operating parameters under heavy loading conditions. The development of optimized coils with larger dimensions might facilitate the accommodation of these animals and the necessary respiration and monitoring accessories. In this work, we present the design, construction and evaluation of a larger transmit/receive birdcage, with optimized parameters, that improved the application range of the 7T scanner by increasing the RF power efficiency, image uniformity and signal-to-noise ratio for relatively large animals.

The proposed hybrid structure was shown to offer a reasonable compromise between efficiency, *B*_*1*_ field distribution, and fabrication complexity. The difficulties to achieve this result tend to increase when the space between the resonator, its shield, and the gradients is reduced [6, 12]. The increased efficiency and SNR were achieved largely through improved matching, but good isolation between channels (< -30 dB) and the well-balanced quadrature (which promoted a homogeneous circularly-polarized excitation field) also contributed.

The final length-to-diameter ratio was 1.13. This value is below the optimal $\sqrt{2}$ recommended for obtaining the highest *B*_*1*_ amplitude at the center of the coil [6]. However, a reduction in RF power demand was obtained with a minor amplitude reduction (around 2%). The smaller length made the coil better adapted to the region of interest (abdomen-thorax), while excluding the legs, head, and other body parts that would otherwise contribute to noise [21].

The segmentation of the legs, which reduces dielectric losses and detuning due to load changes [12], tended to shorten the tuning/matching process between rabbits. In fact, it was found that the coil could be re-tuned between the studied rabbits by a slight adjustment of the matching capacitors. These capacitors are located within the matching network but they also affect the coil resonance, as they change the total reactance of the corresponding end-ring section.

The greatest performance advantage of the proposed coil over the commercial coil will be observed under heavy loading conditions. The most important evidence in support of this assertion was found in the SNR maps computed from the water/NaCl/sugar phantom images. This phantom has a greater loading effect on the coils than the selected group of examined rabbits (approximately 2x) since it is located entirely within the coil, whereas some portions of the rabbits are left outside it. Consequently, further increases to SNR can be expected for larger rabbits (which can weigh up to 5 kg).

A non-uniformity was clearly observed in the mineral oil images. The pattern is the same for both coils, which means that the cause may not be related to them, but probably to the static field due to an imperfect active shimming. This unwanted inhomogeneity could have influenced the uniformity value of the proposed coil, as the maximum intensity value may have been biased by the brighter region.

Good matching under reduced loading conditions, such as the oil phantom, was not achieved with the current coil configuration due to the matching networks, but that was not the objective of this work. For such cases, the available commercial coil can be used.

It was also observed that the SNR in some image areas was lower for the proposed coil; for instance, the entire selected kidney in the GEMS axial image. A closer look at this region reveals that the SNR in the left part of the kidney is similar for both coils and the reduction of the average value is caused by the other parts where the intrinsic, and circularly symmetric, field distribution of the proposed coil creates the darker regions. The less symmetric field distribution of the commercial coil is the cause of these uneven results, as some regions are favored and others are negatively affected.

The use of PCB reduced the component count, fabrication time and total costs, and increased symmetry and reliability, mainly because solder joints and physical components were not present in the legs [12]; however, despite these advantages, the Q of the PCB-based parallel-plate leg capacitors, which is lower than the values typical of ceramic capacitors, leaves room for improvement.

The application range of the coil can be extended to smaller loads in the future by redesigning the matching networks. In addition, the enlarged space inside the coil can be also used to additionally improve image quality. For this purpose, the proposed coil can be provided with decoupling elements, operated in transmit mode and combined with receive-only coils, which currently can be more easily installed, as proposed before [22, 23]. A significant increase in SNR might be expected with this combination, mainly in the external regions of the subjects.

## Conclusions

In conclusion, the proposed birdcage has expanded the possibilities of a preclinical 7T Agilent MRI system, as it allows performing improved image studies on relatively large animals. Large-bodied New Zealand rabbits, valuable in obesity research, can now be studied with reduced RF power demand, increased image uniformity and higher SNR. These achievements have a direct positive impact on the reduction of either the scan time and anesthesia, or the RF deposition, which protects the health of the animals. In addition, the enlarged interior space of the coil facilitates the installation of sensors and ventilation tubes, as well as the incorporation of receive-only coils to improve sensitivity.
